# Australian Podiatry Research in Rheumatology: A Bibliometric Analysis

**DOI:** 10.1002/jfa2.70128

**Published:** 2026-02-02

**Authors:** Shan M. Bergin, Polly Q. X. Lim, Hylton B. Menz, Peta E. Tehan, Matthew R. Carroll

**Affiliations:** ^1^ Discipline of Podiatry, School of Allied Health, Human Services and Sport La Trobe University Melbourne Victoria Australia; ^2^ Department of Surgery, School of Clinical Sciences, Faculty of Medicine, Nursing and Health Sciences Monash University Clayton Victoria Australia; ^3^ School of Allied Health, Faculty of Health and Environmental Sciences Auckland University of Technology Auckland New Zealand

**Keywords:** bibliometrics, podiatry, rheumatology

## Abstract

**Background:**

To conduct a bibliographic analysis of English language foot and ankle research pertaining to rheumatology published by Australian authors.

**Methods:**

The Scopus database search was conducted to identify all Australian rheumatology articles published by podiatric authors in English from 1970 to 2024. Bibliometric analysis was performed using an open‐source tool based on the R language. Citations, journals, authors, institutions and countries were described. Publications were manually categorised according to research type, level of evidence and funding source.

**Results:**

The search strategy yielded 89 eligible articles, which received a total of 2438 citations and were published by 200 authors. The most frequent journals were *Arthritis Care & Research* and *Osteoarthritis and Cartilage* each with 9 articles or 10% of total publications. The most published institution was La Trobe University (affiliation of 151 authors). Most of the Australian rheumatology articles focused on the evaluation of treatments and therapeutic interventions (*n* = 35; 39%) and 11 articles (12%) provided Level I evidence. Forty‐two publications (47%) were supported by Category 1 funding, however, 29 (33%) reported no research funding.

**Conclusion:**

Rheumatology represents just 5% of Australian podiatry research. Despite this, it attracts high citation rates relative to number of publications and is well supported by Category 1 funding in comparison to other research fields. Funding sources outside of competitive Category 1 grants appear to be limited however, and research scope is narrow with a high number of evaluative studies conducted. Rheumatology research would benefit from an increase in available funding sources and a broader research scope that informs disease prevention and evidence‐based clinical care.

## 1 Background

It is estimated that the worldwide prevalence of rheumatic conditions is approximately 5%, with a point prevalence of self‐reported resultant foot problems of 99 per 100 people [1]. Foot and ankle involvement is highly prevalent in rheumatological diseases such as rheumatoid arthritis (RA) [2], osteoarthritis (OA) [3] and gout [4]. Involvement of the small joints of the foot and ankle leads to chronic and acute pain, deformity and functional impairment, putting those affected at increased risk of falls and reduced levels of independence [1, 5, 6].

An estimated 70%–90% of individuals with RA report associated foot and ankle pain with foot involvement associated with a higher incidence of disease activity, reduced function and lower rates of disease remission [7]. Foot OA affects approximately 17% of adults over 50 years of age, with the first metatarsophalangeal joint most commonly affected, resulting in debilitating pain and reduced mobility [8]. Although less prevalent, conditions such as gout, systemic lupus erythematosus, scleroderma and psoriatic arthritis are all associated with one or more of foot pain, deformity, neurological changes and changes to the skin and nails, negatively impacting quality of life [4, 9, 10, 11].

Podiatrists play a critical role in the assessment and management of rheumatological conditions of the foot and ankle. Expertise in biomechanical assessment, dermatological care, orthotic and footwear prescription and wound management contribute to positive improvements in pain and functional outcomes when provided as part of a multidisciplinary approach [12, 13, 14].

Despite the frequency with which rheumatic conditions affect the foot and ankle, this area remains under‐researched when compared with other manifestations of rheumatic disease [6, 7, 15]. Australian researchers have contributed to bridging this research gap through development and recommendations for clinical protocols, validation of assessment tools and interventional studies aimed at mitigating resultant disability [15, 16, 17, 18]. However, continued investment in research, education and service delivery is essential to improve outcomes for affected individuals.

As part of a national research priorities initiative [19], this study sets out to map the landscape of Australian rheumatological research through a comprehensive bibliographic analysis. Our objective was to better understand who was involved in this research, the nature and location of the work being undertaken and the sources of funding support. This information will support the ongoing evolution of rheumatological research in Australia, which is essential to improve outcomes for individuals affected by rheumatic foot and ankle disorders.

## 2 Method

We conducted a bibliometric analysis of articles between January 1970 and December 2023 using data from the Scopus database (Elsevier, Amsterdam, the Netherlands). The Scopus database was chosen due to its wider coverage of journals compared to PubMed and the Web of Science [20, 21]. The study is reported using the bibliometric analysis guidelines suggested by Montazeri et al. [22].

### 2.1 Search Strategy

In line with bibliometric analyses, a broad general search strategy was developed that aligned with key concepts: (i) joint diseases, (ii) deformity, gait, pain and physical function and (iii) foot and footwear. Full details are provided in Supporting Information S1.

### 2.2 Article Selection

The titles and abstracts of all articles were downloaded from the Scopus database and exported into a systematic review application (Covidence, Veritas Health Innovation, Melbourne, Australia). Titles, abstracts and full text articles were then independently screened by two researchers with disagreements resolved by discussion or a third researcher. Eligible publications were original articles or systematic reviews published in English, completed at an Australian education or healthcare institution, in an Australian cohort of participants, where at least one author had an Australian affiliation. For systematic reviews, the first or last author was required to have had an Australian affiliation. Eligible research was deemed to be clinically relevant if it demonstrated applicability to podiatry practice, specifically, if its findings could inform clinical decision‐making or patient care, workforce planning and continuing education. Laboratory‐based studies or pre‐clinical studies were not included as they were not deemed to be directly relevant to podiatry clinical practice. Guidelines, consensus documents, case studies, research letters, editorials, commentaries and conference abstracts were not included.

### 2.3 Data Extraction

Articles were imported into Biblioshiny (*bibliometrix* package version 2.2.1; University of Naples Federico II, Naples, Italy) [23]. The following characteristics were extracted from each article: year of publication, journal name, 2023 Impact Factor (using Journal Citation Reports [Clarivate Analytics, Philadelphia, Pennsylvania, USA]), number of citations (as recorded in the Scopus database [Elsevier, Amsterdam, Netherlands]), author names and institutional and country affiliation.

### 2.4 Data Synthesis

Research type was categorised according to the United Kingdom Clinical Research Collaboration (UKCRC) Health Research Classification System [24]. This system classifies types of research activities using 48 codes within eight groups: (i) underpinning research, (ii) aetiology, (iii) prevention of diseases and conditions and promotion of wellbeing, (iv) detection, screening and diagnosis, (v) development of treatments and therapeutic interventions, (vi) evaluation of treatments and therapeutic interventions, (vii) management of diseases and conditions and (viii) health and social service research. The level of evidence was manually assigned to each study using the National Health and Medical Research Council (NHMRC) criteria [25], which specifies the following: (i) Level I: evidence from a systematic review of all relevant randomised controlled trials. (ii) Level II: evidence from at least one properly designed randomised controlled trial. (iii) Level III: evidence from other well‐designed experimental or analytical studies. (iv) Level IV: evidence from descriptive studies. Funding sources were documented, according to the Australian Government Higher Education Research Data Collection (HERDC) specifications, [26] as follows: (i) Category 1: Australian competitive grant research and development income. (ii) Category 2: other public sector research and development income. (iii) Category 3: industry and other research and development income. (iv) Category 4: cooperative research centre research and development income.

## 3 Results

### 3.1 Article Characteristics

**FIGURE 1:**
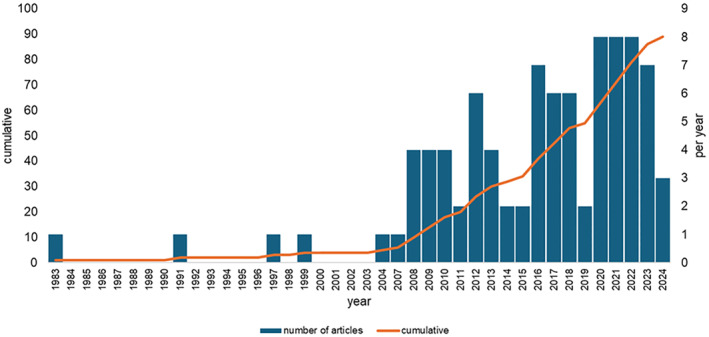
Number of articles (cumulative and per year).

The search strategy yielded 89 eligible articles (see Supporting Information S2). Table 1 presents the characteristics of these articles. The articles received a total of 2438 citations and were published by 200 authors. Table 2 shows that the top 10 most frequent journals were *Arthritis Care & Research*, *Osteoarthritis & Cartilage, Journal of Foot and Ankle Research, Clinical Biomechanics, Gait and Posture, Journal of Orthopaedics and Sports Physical Therapy, Knee, Annals of Internal Medicine, Annals of the Rheumatic Diseases and Arthritis and Rheumatism.* Figure 1 shows the number of rheumatology articles (cumulative and per year) from 1983 to 2023 with the most consistent output occurring between 2020 and 2023.

**TABLE 1:** Publication characteristics.

Years	1983–2023
Total number of articles	89
Original articles	80
Systematic reviews	9
Mean years from publication	8.66
Mean citations per year per article	28.12
Citations	2503
Total authors	199
Mean co‐authors per article	5.6
International co‐authorships (%)	29.21
Single‐authored publications	0

**TABLE 2:** Top 10 most frequent journals.

Journal	*n* (%)	Impact factor 2024
1. *Arthritis* *Care* *&* *Research*	9 (10)	3.3
2. *Osteoarthritis and Cartilage*	9 (10)	9.0
3. *Journal of* *Foot and* *Ankle* *Research*	5 (6)	2.2
4. *Clinical* *Biomechanics*	4 (4)	1.4
5. *Gait and Posture*	4 (4)	2.4
6. *Journal of* *Orthopaedic* *& Sports* *Physical Therapy*	3 (3)	6.3
7. *Knee*	3 (3)	2.0
8. *Annals of Internal Medicine*	2 (2)	15.3
9. *Annals of the* *Rheumatic* *Diseases*	2 (2)	20.6
10. *Arthritis & Rheumatism*	2 (2)	10.9

Abbreviation: IF, impact factor.

### 3.2 Authors, Institutions and Countries

Table 3 displays the top 10 most frequent authors of the articles, with the five most published authors being Menz HB, Munteanu SE, Hinman RS, Bennell KL and Wrigley TV. Table 4 displays the top 10 most frequent institutional affiliations, with the five most frequently represented institutions being La Trobe University, the University of Melbourne, the University of Queensland, Monash University and the University of Western Sydney/Western Sydney University.

**TABLE 3:** Top 10 most frequent authors.

Author	Articles *n*, (%)
1. Menz H.B.	34 (38)
2. Munteanu S.E.	27 (30)
3. Hinman R.S.	26 (29)
4. Bennell K.L.	23 (25)
5. Wrigley T.V.	15 (17)
6. Landorf K.B.	13 (15)
7. Paterson K.L.	12 (13)
8. Metcalf B.R.	11 (12)
9. Auhl M.^a^	10 (11)
10. Roddy E.^a^	10 (11)
11. Tan J.M.^a^	10 (11)

^a^ Equal.

**TABLE 4:** Top 10 most frequent affiliations.

Institution	*n*
1. La Trobe University	151
2. University of Melbourne	113
3. University of Queensland	21
4. Monash University	16
5. University of Western Sydney/Western Sydney University	11
6. University of Newcastle	10
7. University of South Australia	9
8. University of Tasmania	7
9. Prince of Wales Medical research Institute	6
10. Victoria University	6

### 3.3 Citations

Table 5 presents the top 10 most highly cited articles according to total citations and average citations per year. Articles with the highest total citations were a prospective study investigating the use of orthotics to manage plantar pressure and RA pain [27], an RCT to assess the effectiveness of lateral wedge insoles on disease progression in knee OA [28], a cross‐sectional study of physiological risk factors for falls in older people with lower limb arthritis [29], a cross‐sectional study investigating the effects of lateral wedges on frontal plane parameters of the knee [30] and an intervention study investigating immediate effects of laterally wedged insoles in individuals with medial knee OA [31].

**TABLE 5:** Top 10 cited articles (total citations and average citations per article per year).

Authors (year)	Publication title	Journal	Citations (*n*)	Average citations per year
Hodge (1999)	Orthotic management of plantar pressure and pain in rheumatoid arthritis	Clinical Biomechanics	201	7.73
Bennell (2011)	Lateral wedge insoles for medial knee osteoarthritis: 12 month randomised controlled trial	BMJ	160	11.43
Sturnieks (2004)	Physiological risk factors for falls in older people with lower limb arthritis	Journal of Rheumatology	152	7.24
Hinman (2012)	Lateral wedge insoles for medial knee osteoarthritis: effects on lower limb frontal plane biomechanics	Clinical Biomechanics	138	10.62
Hinman (2008)	Lateral wedges in knee osteoarthritis: what are their immediate clinical and biomechanical effects and can these predict a 3‐month clinical outcome?	Arthritis Care & Research	133	7.82
Simic (2013)	Altering foot progression angle in people with medial knee osteoarthritis: the effects of varying toe‐in and toe‐out angles are mediated by pain and malalignment	Osteoarthritis and Cartilage	125	10.42
Kemp (2008)	Reducing joint loading in medial knee osteoarthritis: shoes and canes	Arthritis Care & Research	82	4.82
Hinman (2008)	Effect of length on laterally wedged insoles in knee osteoarthritis	Arthritis Care & Research	77	4.53
Williams (2010)	Feasibility and outcomes of a home‐based exercise programme on improving balance and gait stability in women with lower‐limb osteoarthritis or rheumatoid arthritis: a pilot study	Archives of Physical Medicine and Rehabilitation	70	4.67
Zammit (2008)	Plantar pressure distribution in older people with osteoarthritis of the first metatarsophalangeal joint (hallux limitus/rigidus)	Journal of Orthopaedic Research	70	4.12

### 3.4 Research Types and Level of Evidence

Using the UKCRC criteria, 34 articles (38%) were focused on the evaluation of treatments and therapeutic interventions, 25 articles (28%) were focused on aetiology, 16 articles (18%) were focused on the development of treatments and therapeutic interventions, 5 articles (6%) focused on detection, screening and diagnosis and a further 5 articles (6%) focused on the management of diseases and conditions. Only 1 article (1%) focused on the prevention of disease and conditions and promotion of wellbeing and there were no articles with a focus on underpinning research or health and social care services research. According to the NHMRC levels of evidence, 9 articles (10%) provided Level I evidence, 15 (17%) provided Level II evidence, 51 (57%) provided Level III evidence and 10 (9%) provided Level IV evidence.

### 3.5 Sources of Funding

Twenty‐nine articles (33%) reported no research funding. Of the remaining articles, the most common source of funding reported was from Category 1 (*n* = 42; 47%), whereas the least commonly reported was from category 2 (*n* = 6; 7%).

## 4 Discussion

The objective of this study was to undertake a comprehensive bibliometric analysis of Australian podiatric rheumatological research, examining who conducts it, the types of studies performed, where they are undertaken and how they are funded. Across 89 eligible articles published between 1983 and 2023, we identified the most active researchers and institutions. Our analysis revealed that rheumatology represents only a small fraction of Australian podiatry research, is driven by a limited group of researchers, focuses predominantly on aetiology and development of treatments and therapeutic interventions and is regularly undertaken without reported funding support. These findings highlight both the narrow scope and under‐resourced nature of podiatric rheumatology research in Australia, providing critical insights to guide future research investment, focus and workforce development in this field.

The total yield of rheumatology articles is the third lowest of the nine research streams represented in our larger bibliographic analysis and represents just 5% of research activity relevant to podiatry in Australia [19]. This is concerning given that rheumatological diseases account for 9% of national healthcare expenditure in Australia (approximately AUD$5.7 B p/a) and the significant role of podiatry in early detection and management of disease manifestations affecting the foot and ankle [12, 14, 16, 32]. Also concerning is the lack of research pertaining to the epidemiology of rheumatic disease in First Nations communities, the traditional owners of Australian land represented by a hugely diverse culture comprised of over 100 self‐identified Nations [33]. In the absence of essential baseline data, research that underpins health needs and outcomes, health service access and optimisation of culturally safe management strategies will remain unknown.

When considering other metrics included as part of the broader bibliometric analysis, Australian rheumatology research ranked eighth (from 10) in terms of total citations (2438) with a mean of 27.4 citations per year per article [19]. This is comparable to dermatology which recorded 91 total articles, 2482 total citations and a mean of 27.3 citations per article per year but is significantly less in terms of citations when compared to gerontology which recorded just 8 total articles less (81) but generated 5024 total citations and 62 mean citations per year per article.

Rheumatology research was more collaborative than any other research stream with a mean number of co‐authors per article of 5.6% and 30% of articles co‐authored with international peers. This finding may be reflective of the representation of Australian podiatrists on international research groups such as the International Foot and Ankle Osteoarthritis Consortium [15] and the Outcome Measures in Rheumatology Foot and Ankle Working Group [34]. There were no single authored publications compared to seven in musculoskeletal research and six in workforce/education research [19]. Australian rheumatology research was published across a range of journals with five of the top 10 specific to arthropathy or rheumatic disease. Other journals in the top 10 ranged from publications strongly aligned to foot and ankle research or gait and biomechanics. Notably, 30% of rheumatology publications appeared in high‐ranking journals with associated impact factors up to 20.6 (*Annals of the Rheumatic Diseases*).

In all included research streams except for rheumatology, First Nations and gerontology, there was a significant discrepancy between the number of studies supported by Category 1 funding and the number of studies conducted with no funding. For example, in neurovascular research, just 8% of research was supported by Category 1 funding with 79% of studies conducted with no funding. In rheumatology, 42 studies (47%) were supported by Category 1 funding with 29 (33%) conducted with no funding support, a much more equitable split. The remaining 18 studies were conducted using either Category 2 or 3 funding. It is difficult to make funding comparisons with other bibliometric analyses as these are either specific to a single rheumatic condition [35, 36] or do not include funding as part of their analysis [37, 38]. It is worth noting that several of the most cited authors in rheumatology research had some access to funding (67% of all publications) and were based in academic institutions, which likely increased their capacity to contribute to more publications from 2008. However, despite this reasonable level of funding, rheumatology research has produced relatively low levels of evidence. Using the NHMRC levels of evidence, only 11 articles (12%) provided Level I evidence, with 15 (17%) providing Level II evidence, 51 (57%) providing Level III evidence and 12 (14%) providing Level 4 evidence.

These findings should be interpreted with several limitations in mind. First, our analysis excluded guidelines, consensus statements, case studies, research letters, editorials, commentaries and conference abstracts and therefore does not capture the full breadth of rheumatology‐related output. Second, assigning articles to a single research category is inherently challenging, and some degree of misclassification is possible. Third, reporting of funding is often inconsistent and does not reliably distinguish between project support and salary funding, meaning the true financial investment in this research area may not be accurate. Finally, citation metrics, while widely used, do not necessarily reflect the societal relevance or real‐world impact of research.

In summary, our bibliometric analysis highlights that rheumatology comprises a very small proportion of Australian podiatry research, and despite reasonable access to funding, it produces a small percentage of high‐level evidence. The strengths of Australian rheumatological research appear to lie in national and international opportunities for collaboration and publication outputs in high impact journals. Ongoing strategic collaboration to maximise the use of available funding is crucial for the growth of rheumatology research in podiatry and to enhance the evidence base for mitigation of associated disability. These findings provide a foundation for national research priority‐setting to strengthen the quality, impact and clinical relevance of Australian podiatry research.

## Author Contributions

**Shan M. Bergin:** conceptualization, funding acquisition, writing – original draft, writing – review and editing. **Polly Q. X. Lim:** data curation, writing – review and editing. **Hylton B. Menz:** conceptualization, funding acquisition, writing – review and editing. **Peta E. Tehan:** conceptualization, project administration, methodology, formal analysis, writing – review and editing. **Matthew R. Carroll:** conceptualization, methodology, formal analysis, writing – review and editing.

## Funding

This study was funded by the Australian Podiatry Education and Research Foundation.

## Ethics Statement

The authors have nothing to report.

## Consent

The authors have nothing to report.

## Conflicts of Interest

The authors declare no conflicts of interest.

## Permission to Reproduce Material From Other Sources

The authors have nothing to report.

## Supporting information


Supporting Information S1

Supporting Information S2

## Data Availability

Data are available from the authors on reasonable request.
