# Supplementary material for: Australian Podiatry Research in Rheumatology: A Bibliometric Analysis
Source: J Foot Ankle Res. 2026 Feb 2;19(1):e70128. doi: 10.1002/jfa2.70128 (PMC12864540; doi:10.1002/jfa2.70128)
Supplement: Supplementary file 1 — Supporting Information S1 [file JFA2-19-e70128-s002.docx]

| 1. rheum*  2. arth*  3. gout*  4. Psoriatic  5. osteo*  6. seronegative  7. “Joint diseases”  8. Gait  9. Plantar pressure  10. Balance  11. Falls  12. “Foot ortho*”  13. Pain  14. Function  15. Deformity  16. Footwear  17. "lower extremity"  18. foot  19. feet  20. Ankle  21. Knee  22. hip  23. pod*  24. AND NOT  25. diabet*  26. diabetes | |
| --- | --- |
| Search Strategy  1: 1 OR 2 OR 3 OR 4 5 OR 6 OR 7 OR 8 OR 9  2: 10 OR 11 OR 12 OR 13 OR 14 OR 15 OR 16 OR 17 OR 18  3: 19 OR 20 OR 21 OR 22 OR 23 OR 24 OR 25  4 1 AND 2 AND 3  5 AND NOT 26 OR 27 | |
| Search Restrictions | |
| Year | 1970-2023 |
| Language | English |
| Source | Article |
| Author affiliation | Australian |
